# Progress in Genome Editing Technology and Its Application in Plants

**DOI:** 10.3389/fpls.2017.00177

**Published:** 2017-02-14

**Authors:** Kai Zhang, Nadia Raboanatahiry, Bin Zhu, Maoteng Li

**Affiliations:** ^1^Department of Biotechnology, College of Life Science and Technology, Huazhong University of Science and TechnologyWuhan, China; ^2^Hubei Collaborative Innovation Center for the Characteristic Resources Exploitation of Dabie Mountains, Huanggang Normal UniversityHuanggang, China

**Keywords:** Genome editing technology, ZFN, TALEN, CRISPR-Cas9, CRISPR-Cpf1, CRISPR-C2c1, RNAi, CRISPR-C2c2

## Abstract

Genome editing technology (GET) is a versatile approach that has progressed rapidly as a mechanism to alter the genotype and phenotype of organisms. However, conventional genome modification using GET cannot satisfy current demand for high-efficiency and site-directed mutagenesis, retrofitting of artificial nucleases has developed into a new avenue within this field. Based on mechanisms to recognize target genes, newly-developed GETs can generally be subdivided into three cleavage systems, protein-dependent DNA cleavage systems (i.e., zinc-finger nucleases, ZFN, and transcription activator-like effector nucleases, TALEN), RNA-dependent DNA cleavage systems (i.e., clustered regularly interspaced short palindromic repeats-CRISPR associated proteins, CRISPR-Cas9, CRISPR-Cpf1, and CRISPR-C2c1), and RNA-dependent RNA cleavage systems (i.e., RNA interference, RNAi, and CRISPR-C2c2). All these techniques can lead to double-stranded (DSB) or single-stranded breaks (SSB), and result in either random mutations via non-homologous end-joining (NHEJ) or targeted mutation via homologous recombination (HR). Thus, site-directed mutagenesis can be induced via targeted gene knock-out, knock-in, or replacement to modify specific characteristics including morphology-modification, resistance-enhancement, and physiological mechanism-improvement along with plant growth and development. In this paper, an non-comprehensive review on the development of different GETs as applied to plants is presented.

## Introduction

Genome editing is a conventional method that is often applied to alter the genotype and phenotype of organisms. In general, two methods are typically adopted for gene function analysis. One of these is the traditional forward genetics method (i.e., moving from phenotypic to genetic changes) which enables the identification of new functional genes via T-DNA tag or map-based cloning. This method involves cultivation and screening for special mutants with key traits (Page and Grossniklaus, 2002). The second of the two methods is reverse genetics (i.e., moving from genetic to phenotypic changes), and involves the identification of candidate genes with obvious differential expression. This approach requires that tissues and cells in different environments are identified by gene chip or bioinformatics so that gene function can be analyzed using genetic transformation technology, including over-expression and knock-out (Takahashi et al., 1994). Conventional genome editing technologies (GETs) including natural mutations via hybridization and induced mutations due to physical factors such as x-rays (Stadler, 1928a,b) and fast neutrons (Li et al., 2001; Wu et al., 2005) or chemical factors such as ethylmethanesulfonate (Wu et al., 2005; Wang et al., 2008), sodium azide (Talamè et al., 2008), and diepoxybutane (Suzuki et al., 2008) offer a variety of new materials for the generation of either autogenous or non-autogenous random variation. Such variants can include point mutations, deletions, translocations, and chromosome losses. In addition, a range of technologies, including protoplast fusion via polyethylene glycol (PEG; Kao and Michayluk, 1974), electronastic stimulus, or sendai virus-induced methods (Salts et al., 1985), have also been widely applied as they are efficient for hybridization, enforcing inheritance from two parents. However, although these techniques are able to alter genome sequences to some degree, they cannot satisfy the requirements of targeted genome modification.

Due to the development of new approaches, current GETs are able to induce double-stranded breaks (DSBs) which either promote random mutations via error-prone non-homologous end-joining (NHEJ; Gorbunova and Levy, 1999) or targeted mutations via error-free homologous recombination (HR; Symington and Gautier, 2011). Thus, sequence deletions, insertions, duplications, replacements, and translocations are frequently observed (Brunet et al., 2009; Garneau et al., 2010; Lee et al., 2010, 2012; Friedland et al., 2013; Xiao et al., 2013; Xie et al., 2014). HR, however, only plays a role in late S/G2 phases, while NHEJ can be applied over all phases of the cell cycle (Sonoda et al., 2006). Thus, although HR is favored for the insertion of an exogenous DNA donor at a DSB for specific genome modifications, it is inefficient compared to NHEJ. Theoretically, NHEJ includes both Ku-dependent and Ku-independent NHEJ pathways (Symington and Gautier, 2011); the former is often referred to as “classical” NHEJ, able to induce several nucleotide insertions or/and deletions with the participation of Ku70/80 proteins, while the latter is capable of inducing a larger number of deletions via microhomology-mediated end-joining (Fattah et al., 2010).

An appropriate method for gene knock-out that was both simple and practical was not available for many years. However, since 1998, RNA interference (RNAi) has been used to provide a rapid, low-cost, and high-throughput technology for the analysis of gene function (Fire et al., 1998). Knockdown using RNAi, however, just makes a DNA sequence diversify, and cannot achieve either permanent or complete gene knock-out (Sachse et al., 2005; Marine et al., 2012). Over the last 10 years, a series of new GETs, including ZFN and TALEN, have become fashionable because they are able to knock-out or modify specific targeted sequences. These GETs are based on chimeric proteins composed of sequence-specific DNA binding domains and non-specific DNA cleavage nucleases (Townsend et al., 2009; Mahfouz et al., 2011; Zhang et al., 2013). Nevertheless, the design of a novel protein that is able to recognize specific DNA is complicated; thus, the adaptive immune system (CRISPR-Cas) have been developed and widely applied for their powerful genome-editing capabilities.

To date, two classes of CRISPR-Cas systems have been developed that differ in the configuration of their effector modules, which was originally classified into several types on the basis of their Cas compositions (Makarova et al., 2015). Class 1 system possessing multi-subunit crRNA-effector complexes. This class encompasses type I and type III as well as a putative type IV, while the class 2 system is characterized by the presence of an effector complex that consists of a single, large Cas protein. This second class encompasses type II and type V as well as type VI (Shmakov et al., 2015; Wright et al., 2016). To date, more attention has been focused on class 2 CRISPR-Cas systems because of their flexible compositional construction that means they can be harnessed to create powerful genome editing tools. These systems enable simple, specific, and economical technology via a RNA-guided process (Cong et al., 2013; Jiang et al., 2013; Jinek et al., 2013; Mali et al., 2013a; Abudayyeh et al., 2016; Kim et al., 2016). Indeed, of the available class 2 systems, CRISPR-Cas9 from the type II CRISPR-Cas system is a RNA-guided DNA cleavage system that has been developed into a powerful GET with high on-target efficiency (Cong et al., 2013). At the same time, CRISPR-Cpf1 (CRISPR from *Prevotella* and *Francisella 1*), type V-A protein, and CRISPR-C2c1 (class 2 candidate 1), a type V-B protein, have also provided active RNA-guided DNA cleavage systems (Shmakov et al., 2015; Kim et al., 2016), while CRISPR-C2c2 (class 2 candidate 2), type VI-A CRISPR-Cas system, has also shown great potential for the targeting and editing of single-stranded RNA (Abudayyeh et al., 2016).

Very recently, a novel but controversial GET of *Natronobacterium gregoryi* Argonaute (NgAgo) has also been developed that is able to break down the limitation of selected target sequences, the secondary structure of single guided RNA, and the conformation of target DNA. Building on these developments, the aim of this paper is to emphasize an non-comprehensive review on different GETs that have been developed in recent years as well as their incomplete applications to plants.

## Development and comparisons of gets

GETs could be divided into two groups. The members of one group are mediated by protein-guided nuclease, such as ZFN and TALEN, while members of the other are mediated by special RNA/DNA-guided nuclease, including RNA-dependent DNA cleavage systems like CRISPR-Cas9, CRISPR-Cpf1, and CRISPR-C2c1, and RNA-dependent RNA cleavage systems like RNAi and CRISPR-C2c2.

## Protein-dependent DNA cleavage systems

**Figure 1:**
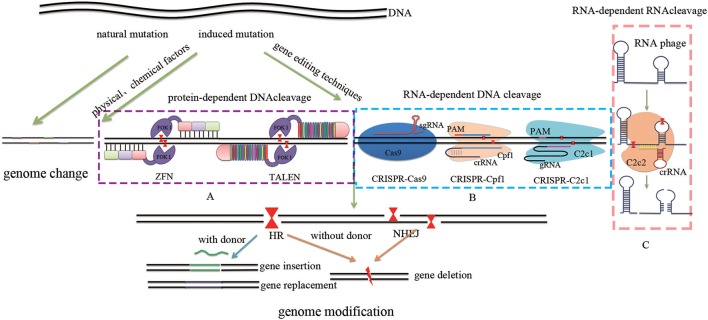
**Comparison of different GETs**. Traditional methods include natural mutation via hybridization, induced mutation via ultraviolet light and x-ray (physical methods), as well as the use of benzene analogs and nitrous acid (chemical methods). These approaches offer a range of raw materials for evolution by randomly generating either autogenous or non-autogenous variation. Site-specific genome targeting technologies such as protein-dependent DNA cleavage systems **(A)** including ZFN and TALEN, as well as RNA-dependent DNA cleavage systems **(B)** including CRISPR-Cas9, CRISPR-Cpf1, and CRISPR-C2c1 can induce DSBs. In contrast, RNA-dependent RNA cleavage systems **(C)** such as SSB give rise to either random mutations via error-prone NHEJ or targeted mutations via error-free HR. These approaches achieve genome modification by inserting, deleting, or replacing targeted DNA sequences.

In 1994, Kim and Chandrasegaran developed the first generation of artificial restriction endonucleases by linking the Ultrabithorax homeodomain of *Drosophila melanogaster* to the cleavage domain of the FokI restriction endonuclease of a *Flavobacterium okeanokoites* (Kim and Chandrasegaran, 1994). Two years later, ZF polypeptides were developed as an alternative to this homeodomain (Kim et al., 1996); the ZF binding domain generally comprises of four-to-six Cys2–His2 arrays which are derived from a transcription factor in human cells. Thus, each ZF is composed of ~30 amino acids arranged in a ββα configuration and mediated by Zn^2+^ (Beerli and Barbas, 2002). Several variable amino acids on the surface of the α-helix at fixed locations are able to bind exclusively to three successive bases in the DNA major groove (Pabo et al., 2001). Therefore, a functional artificial ZFN can be constructed by fusing the ZF binding domain with a restriction enzyme, FokI, extracted from *F. okeanokoites*, which is active as dimers (Figure 1A). The key to recognizing specific DNA is the presence of a five-to-seven base pair (bp) spacer between two ZFN target sites that provides a microenvironment for the FokI cleavage domain. The use of ZFNs has proven an optimal strategy for efficient and precise genome editing as these can induce DSBs at a targeted location (Kim et al., 1996; Figure 1A). At the same time, however, difficulties inherent to designing ZF binding domains that perfectly match triplet codes as well as the context-dependent nature of interactions with neighboring amino acids have restricted the utilization of this approach (Maeder et al., 2008).

Building on this, a further protein-dependent DNA cleavage platform, TALEN, was listed as a breakthrough of the year in *Science* 2012. This platform, TALE (or AvrBs3/PthA), was discovered in the plant pathogenic bacteria *Xanthomonas* Hrp-type III secretion (T3S) system (Boch and Bonas, 2010); similarly, linking with FokI enables the introduction of DSBs within site-specific sequences. The TALE comprises three parts, one of which is composed of 13.5–25.5 tandem repeats (i.e., the last repeat is half of a unit in the 3′ terminal) that enables specificity of targeted gene recognition. Each of these repeats usually contains 34 amino acid motifs which are almost identical with the exception of two in positions 12 and 13. This section is referred to as a repeat-variable diresidue (RVD) as it is able to recognize and bind regularly to A, C, T, and G. In contrast, the other parts of TALE include N-terminal and C-terminal sequences (Boch et al., 2009; Moscou and Bogdanove, 2009; Figure 1A). As a result of this composition, single base recognition between TALE and DNA-binding repeats is more flexible than ZF proteins which are triplet-limited. At the same time, however, precise recognition by TALE requires a special RVD to identify 5′ methylation C which is sensitive to the target DNA sequence (Boch et al., 2009). In addition, construction of a TALE array presents a technical challenge because of widely identical repeat sequences. More than 1,000 amino acids are required to identify just 20 bases, a process which may lead to an immune response and reduce the efficiency of target recognition (Sander et al., 2011).

Most recently, “base editor”-targeted deaminase technology has been applied to ZF and TALE approaches in order to enhance efficiency and accuracy. Cytosine deaminase, is an enzyme absent in mammalian cells but present in microbes and fungi Building on this observation, Austin and Huber (1993) were the first to clone, sequence, and express *Escherichia coli*. cytosine deaminase and apply it as a gene therapy for the treatment of metastatic colorectal carcinomas (Austin and Huber, 1993). Subsequent research showed that apolipoprotein B editing complexes (APOBECs) and activation-induced deaminase (AID) are also kinds of cytosine deaminase that are able to induce RNA and/or DNA mutations and play a role in the immune system (Muramatsu et al., 1999; Conticello, 2008). These enzymes have been shown to be able to convert cytidines (C) into uracils (U) which themselves are treated as thymine (T). This leads to C = G to T = A conversion in DNA strands unable to induce HR or NHEJ repair mechanisms (Rada and Di Noia, 2004). Yang L. et al. (2016) went further and fused cytosine deaminases to ZF and TALE binding domains, respectively, increasing site-specific C → T conversions by 13% in *E. coli* and 2.5% in human cells. Compared to ZF-APOBECs, ZF-AID possessed a targeted deaminase frequency with highest GFP by correcting ACG to ATG, while this efficiency for TALE-AID fell between ZF-APOBECs and ZF-AID. Almost no off-target conversions were observed when this approach was applied, although several unintended off-target WRC (W = A/T, R = A/G) motifs did result. In addition, research has shown that the cytotoxicity of targeted deaminase ZF-AID is less than is the case for ZFN (Yang L. et al., 2016).

In sum, ZFN and TALEN are composed of DNA-binding protein and the enzyme FokI, both require the elaborate construction of individual DNA-binding proteins for each DNA target site (Zhang et al., 2010; Li et al., 2012).

## RNA-dependent DNA cleavage systems

The CRISPR-Cas series presently provides a robust RNA-dependent DNA cleavage system. Because this approach is based on the adaptive immunity system, widely existing in bacteria and archaea to enable resistance to virus or plasmid invasion, thus showing extreme diversity in Cas protein compositions, genomic loci architecture, and defense mechanisms (Makarova et al., 2011, 2015).

**Figure 2:**
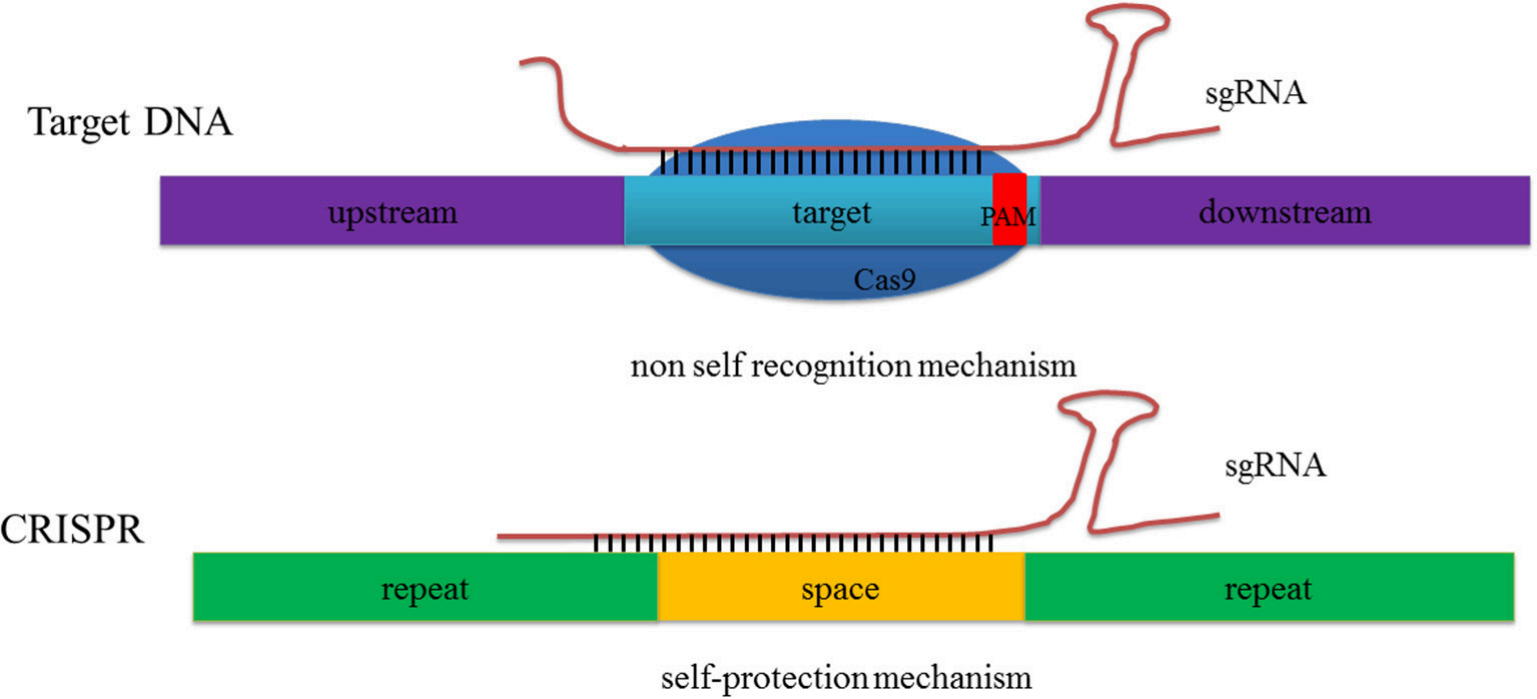
**The CRISPR/Cas9 system does not recognize itself but target DNA**. Both the 8–9 bp repetitive sequence of 5′ end crRNA and the neighbor spacer can match the CRISPR loci of itself while at the same time just the target repeat sequence match can guide the Cas9 to induce target DNA cleavage.

The basic mechanisms of the CRISPR-Cas system-mediated acquired immune system can be roughly divided into three stages (Wiedenheft et al., 2012; Hille and Charpentier, 2016). In the first, adaptation, bacteria and archaea incorporate short sequences from invading genetic elements (i.e., a virus or plasmid) into their genomes. In the second, expression, invading sequences are transcribed and processed into pre-crRNA (precursor CRISPR RNA) under the regulation of leader sequences. When a similar exogenous nucleic acid invades a second time, pre-crRNA sequences can then be further processed into shorter mature crRNA. In the third stage, interference, a tracrRNA-crRNA complex is formed by base pairing and acts to guide the effector protein in recognizing and cleaving the invading foreign DNA sequence homologous to the spacer. Additional research has even elucidated the reason why the CRISPR-Cas system cannot recognize DNA on its own, but just targets sequences; Wiedenheft et al. (2012) argued that this system benefits from the protospacer adjacent motif (PAM) region, and an eight-to-nine bp repetitive sequence in the 5′-end of crRNA that matches the spacer in the CRISPR loci prevent this protein from self-cutting (Wiedenheft et al., 2012; Figure 2).

To-date, three RNA-dependent DNA cleavage systems have been reported.

## The CRISPR-Cas9 system

The most powerful GET currently, CRISPR-Cas9, was first reported by Jinek et al. (2012). This system is able to recognize a specific site in a target gene with high efficiency, specificity, and flexibility (Jinek et al., 2012; Figure 1B). The CRISPR-Cas9 system comprises two main components, one of which is called CRISPR RNA (crRNA), transcribed from interval spacer sequences that correspond to sequences on the phage or plasmid (prospacer). This sequence consists of highly conserved repeats and markedly different interval spacers that are homologous to the prospacer from the exogenous phage and plasmid (Mojica et al., 2005). The main function of crRNA is to match and recognize target DNA, while another component of this system is Cas protein, close to CRISPR array and responsible for the cleavage of target DNA (Ishino et al., 1987). The numbers of CRISPR as well as its repetitions are highly variable in different species, accounting for the observed diversity in defense functions (Wei et al., 2013). In general, the 5′ or 3′ end of the protospacer has several extended conserved bases, which are called PAMs. Each PAM is between two and five bases in length and is located one-to-four bases away from the protospacer; the composition of each PAM commonly comprises NGG, NRG (R equals G or A) could also be observed in some cases (Hsu et al., 2013; Jiang et al., 2013). Indeed, the one-to-five bp sequence proximal to each PAM in crRNA might determine cleavage specificity and is called the “seed sequence” (Duan et al., 2014; Wu et al., 2014). In contrast, the distal sequences of PAMs are called “non-seed sequences” and may take part in targeting cleavage by triggering conformational changes in Cas9 (Cencic et al., 2014).

The current dominance of the CRISPR-Cas9 approach is the result of several landmark studies that were published at the beginning of 2013 (Cong et al., 2013; Jinek et al., 2013; Mali et al., 2013b). This approach has proved efficient because the Cas9 enzyme has two active centers (RuvC and HNH) that enable strong cutting ability of double-stranded DNA (dsDNA), guided by crRNA, and trans-activating crRNA (tracrRNA). The 5′ end of tracrRNA linked to the 3′ conserved and extended sequence of crRNA may form a hybrid molecule by base pairing, and assists with the formation of a protein-RNA complex by its special space structure with Cas9. This means that this approach is able to further match targeted DNA and achieve DSB by the presence of 20 upstream bases relative to the PAM at the 5′ end. Indeed, the longer the chain on the tracrRNA 3′ end, the better the effect of Cas9 (Hsu et al., 2013). Jinek et al. (2012) creatively merged crRNA with a partial tracrRNA into a chimeric RNA chain and showed that the functions of both can be retained simultaneously (Jinek et al., 2012). As discussed, the chimeric RNA chain dual-tracrRNA:crRNA, which joins crRNA and tracrRNA together into a ring connected by four bases, is called single guide RNA (sgRNA). This is important because only when guided with an appropriate sgRNA, can Cas9 successfully cleave targeted DNA (Jinek et al., 2012). It has even been reported that optimizing the length of an sgRNA sequence may increase on-target efficiency; Fu et al. (2014), for example, reported that a sequence between 17 and 18 nt in length can decrease off-target effects 5,000-times or more without sacrificing on-target efficiency. These authors also speculated that the off-target ratio could be further reduced by increasing GC contents (Fu et al., 2014).

Subsequent to synthesis of Cas9 and gRNA, targeted gene editing can be achieved via delivery into tissues or cells. However, the method utilized to deliver a vector into an animal or plant cell varies a great deal. In animals, for example, a vector containing gRNA and Cas9 can be injected straight into gametes or zygotes. This procedure was pioneered by Cong et al. (2013) who set a procedure for site-specific gene editing in human cells (Cong et al., 2013); this approach is now widely and successfully applied across all kinds of animals. In the case of plants, PEG-mediated transfection to protoplasts (Li et al., 2013; Liang et al., 2014), *Agrobacterium*-mediated transformation to the leaf and embryo (Nekrasov et al., 2013; Upadhyay et al., 2013), and particle bombardment using a gene gun (Miao et al., 2013; Li et al., 2015) are all widely used methods that have led to positive results. Additional research has revealed that codon optimization, as well as the refit of promoters and the number of NLS in Cas9 and sgRNA can affect the efficiency of site-directed delivery (Mikami et al., 2015). For example, the use of either one or two NLS when targeting young seedling albino (YSA) alongside the promoters *Os*U3 and *Os*U6 generates 53.3 vs. 39.6% and 75 vs. 80% mutation rates, respectively (Mikami et al., 2015). The use of codon-optimized Cas9 combined with *Os*Cas9 enables 77.1% on-target efficiency while none were detected when using *At*Cas9 (Mikami et al., 2015). Recently, use of a novel CRISPR/Cas9 vector, pKAMA-ITACHI Red (pKIR), optimized with the ribosomal protein S5A (RPS5A), a Cas9 promoter, enabled both high-efficiency and heritable mutations in *Arabidopsis* (Tsutsui and Higashiyama, 2017). Further, use of the RPS5A optimized-promoter for Cas9 was efficient in 66.7% of albino second leaves in T1 plant cotyledons, while 6.1 and 3.1% were seen, respectively, when WOX2 and 35S optimized-promoters were used to target the PDS3 gene.

When Cas9 and gRNA have been delivered into cells and activated, regulatory mechanisms must be in place to restrain or stop these systems from just carrying out limited knock-out of target genes. To do this, two distinct families of anti-CRISPR associated proteins were identified and used for type I (Pawluk et al., 2016a), while recently a new robust, specific, and genetically encodable “off-switch” for Cas9 activity has been identified (Pawluk et al., 2016b). These authors also identified three completely unrelated sequences of anti-CRISPR families (i.e., *acrIIC1*_Boe_ and *acrIIC1*_Nme_, *acrIIC2*_Nme_, and *acrIIC3*_Nme_) that can bind directly to the NmeCas9/sgRNA complex and prevent *in vitro* DNA cleavage. Pawluk et al. (2016b) also showed 50% cleavage inhibition when the anti-CRISPR:NmeCas9 ratio was held at 1:1, while complete inhibition was observed at 5:1. A striking decrease in the ability of NmeCas9 to create genome lesions was observed when these anti-CRISPR members were tested in human HEK293T cells (Pawluk et al., 2016b).

### Design of Cas9 for genome editing

The best-studied Cas endonuclease, Cas9, is a multi-functional protein that has a molecular weight of 160 kDs in type II CRISPR-Cas system. This endonuclease has two structural domains: HNH is responsible for cleaving complementary DNA (cDNA) three nts adjacent to the PAM, while its counterpart, the RuvC-like domain, cleaves strands of non-cDNA in a three-to-eight nt region adjacent to the PAM (Friedland et al., 2013; Xie et al., 2014). Several approaches are available for on-target optimization.

**Figure 3:**
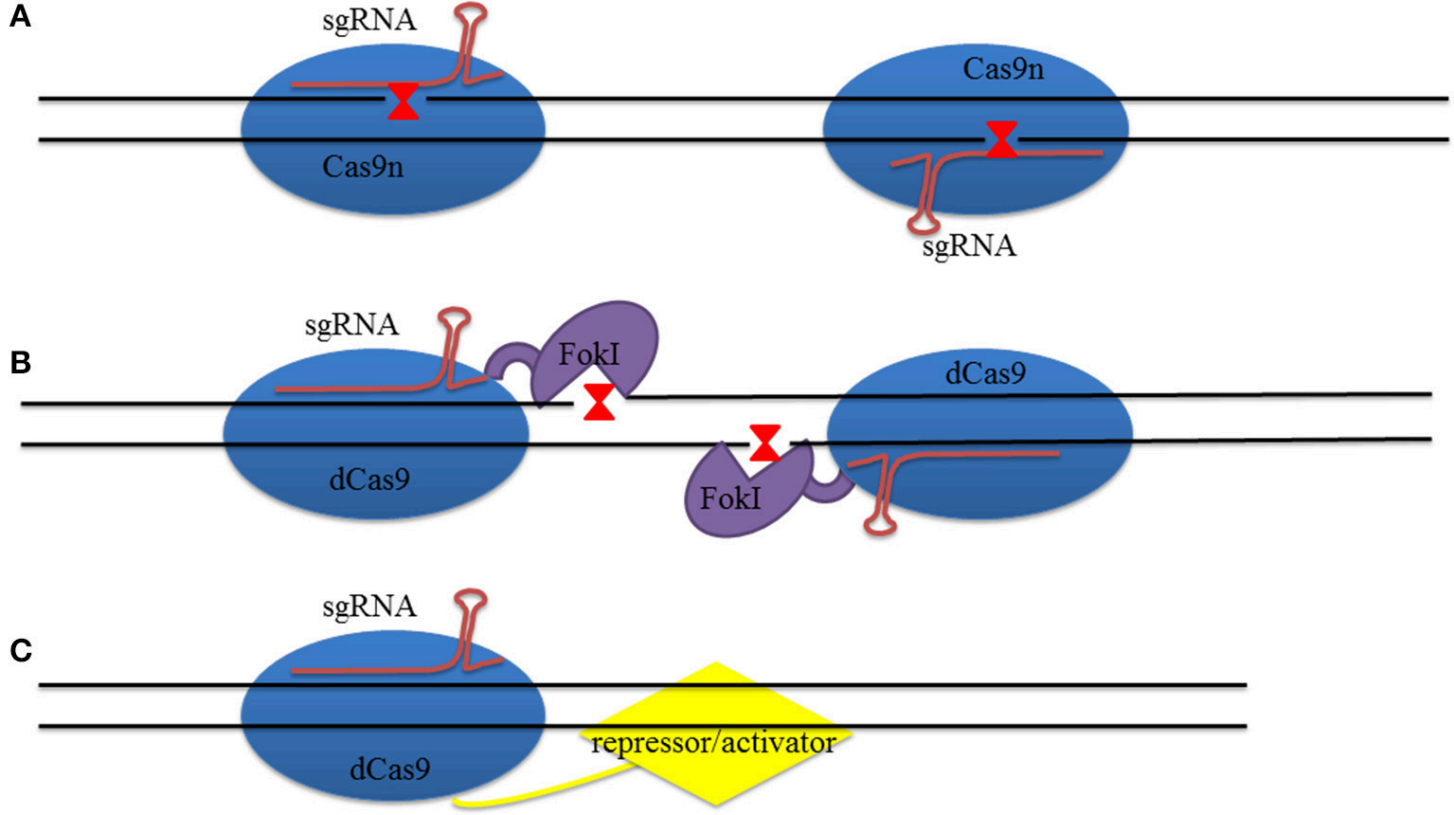
**Design of Cas9 for genome editing. (A)** Two Cas9ns guided with sgRNAs reduce SSBs. When two SSBs are adjacent to one another they generate a DSB which increases the on-target specificity. **(B)** Catalytically-inactive Cas9 (dCas9) protein fused with FokI nuclease to decrease off-target events. **(C)** dCas9 loaded with inhibiting factors, or activators, to repress, or activate, gene transcription.

However, an off-target problem will still exist even if this system exhibits outstanding superiority compared to site-specific GET. Thus, researchers have expended large amounts of effort to increase on-target efficiency and decrease the number of off-target events. For example, it has been shown that Cas9 endonuclease can be mutated to Cas9 nickase (Cas9n) that can only induce SSB when RuvC D10A or HNH H840A mutation is introduced (Figure 3A). The number of off-target events was decreased more than 50 times when these two Cas9ns were combined with two sgRNA sequences. This is because when these two sequences are present at the same time, the number of mismatches decreases while higher precision HR increases with double Cas9ns (Ran et al., 2013). At the same time, the fCas9 (FokI-dCas9, deactivated Cas9 that has mutations on both the D10A gene of RuvC and the H840A gene of HNH) complex can also be used to reduce the off-target ratio (Figure 3B); this approach improved site-specific targeting by fusing FokI with inactivated Cas9 endonuclease, while on-target specificity requires two fCas9 monomers to bind together. The specificity of these modified fCas9s can be up to 140 times higher than use of just wild type Cas9 in human cells (Guilinger et al., 2014). Research has also shown that the use of RNA-guided FokI nuclease (RFN) dimers can also be helpful for reducing the off-target ratio. This ratio was lower when single gRNA guided RFNs were used compared to just Cas9n; in addition, the use of dimeric RFNs can greatly increase on-target efficiency when multiple gRNAs are expressed at the same time and are not restricted by PAMs (Tsai et al., 2014).

Optimized CRISPR ‘base editor’-targeted deaminase technology-has also recently been developed, which enables efficient point mutations rather than just random insertions or deletions (indels). This approach also has the advantage that it does not generate DSBs by fusing targeted deaminase with dCas9 guided by sgRNA (Hess et al., 2016; Komor et al., 2016; Ma et al., 2016). Hess et al. (2016) developed this novel approach and performed a mutation in the base region between 12 and 32 bp downstream of the PAM, while Ma et al. (2016) targeted the region between 12 and 16 bp upstream of the PAM (Hess et al., 2016; Ma et al., 2016). In addition, Hess et al. (2016) were able to successfully alter wild type GFP to brighter EGFP by directed mutation of S65T and F64L residues, obtaining a series of bortezomib-resistant PSMB5 mutants (Hess et al., 2016), while Ma et al. (2016) identified imatinib-resistant mutants by targeting the BCR-ABL gene (Ma et al., 2016). Komor et al. (2016) fused dCas9 with four different cytidine deaminases (i.e., human AID, human APOBEC3G, rat APOBEC1, and lamprey CDA1) and showed that rat APOBEC1-dCas9 had the highest conversion efficiency, as it linked with more than nine amino acids (i.e., BE1, APOBEC1-XTEN-dCas9). In comparison with BE1, the editing efficiency of BE2 (i.e., APOBEC-XTEN-dCas9-UGI) is three-fold higher; both, however, were able to decrease indel rates to <0.1% in human cells. Encouragingly, the base editing efficiency of BE3 (i.e., APOBEC-XTEN-dCas9 (A840H)-UGI) was even higher, an increase of between two and six times compared to BE2 with almost no indel (Komor et al., 2016). These results demonstrate that fusing cytidine deaminase with the CRISPR-Cas system presents a powerful approach for studying and improving gene and protein functions in the future.

## The CRISPR-Cpf1 system

The cas gene cpf1 was first identified in the *novicida* strain U112 of a subspecies of *Francisella tularensis* (Schunder et al., 2013) and later in other bacteria of this genus as well as in the archaean *Prevotella* (Schunder et al., 2013; Vestergaard et al., 2014; Zetsche et al., 2015). The CRISPR-Cpf1 system, classified as type V-A within class 2, is composed of an ordered cpf1-cas4-cas1-cas2-CRISPR array. Of these, cas1 and cas2 proteins act as adaptation modules involved in spacer DNA acquisition just as in other CRISPR-cas systems (Makarova et al., 2011). In contrast, Cpf1, a characteristic effector module seen in type V-A, is a large protein of about 1,300 amino acids that can be subdivided into a N-terminal helical, a central oligonucleotide-binding domain (OBD), and a C-terminal RuvC domain (Zetsche et al., 2015; Dong et al., 2016). In contrast to the Cas9 system, the N-terminal portion of Cpf1 contains a mixed α/β helical structure, while mostly α-helical recognition lobes are seen in the N-terminal of cas9 (Zetsche et al., 2015). Cpf1 only has a RuvC-like endonuclease domain but lacks a HNH endonuclease domain; thus, the three parts of Cpf1 form a bilobal, triangle-shaped architecture with a large, positively-charged channel in the center (Dong et al., 2016). Research by Dong et al. (2016) has shown that the crystal structure of the full-length LbCpf1 in complex with crRNA is at 2.38Å resolution (Dong et al., 2016). Upstream to the crRNA, PAM loci are located within a 5′-end T-rich region (5′-TTN); Zetsche et al. (2015) showed that of these, the middle T is more important than the first T (Zetsche et al., 2015).

The CRISPR-Cpf1 system is a minimalistic, but functionally adaptive defense system (Figure 1B) that was first developed as a robust GET in human cells (Zetsche et al., 2015) and has now been successfully applied in rice (Xu et al., 2016). The constituent Cpf1 is a dual-nuclease, specifically for processing pre-crRNA to mature crRNA, that comprises 19 nt DR fragments followed by a 23–25 nt spacer sequence. In contrast to Cas9, this nuclease does not require tracrRNA or RNaseIII and can also cleave target DNA and introduce DSBs with a crRNA guide consisting of a single stem loop in the DR sequence (Zetsche et al., 2015; Fonfara et al., 2016). Initially, it was thought that Cpf1 forms a homodimer to target DNA cleavage, but later crystal structure studies have revealed that the OBD of this nuclease recognizes crRNA in a highly-distorted conformation. At the same time, the 3′ end of crRNA is directed into the central channel of Cpf1 where extensive intramolecular interactions take place (Dong et al., 2016). Dong et al. (2016) speculated that the octahedral (Mg(H_2_O)6)^2+^ ion may play an important role in stabilizing the conformation of crRNA and further enhancing Cpf1 recognition (Zetsche et al., 2015; Dong et al., 2016). The cleavage site is known to occur at, or after, the 23rd nt on the target strand and after the 18th nt on the non-target strand; thus, the length of the 5′ overhang can range between two and five nt and further augment the NHEJ repair mechanism (Zetsche et al., 2015; Kim et al., 2016). The DR portion of mature crRNA should therefore be at least 16 nt in length, and between 17 and 18 nt to maximum cleavage efficiency. Subsequent research has shown that the stem-loop duplex structure of crRNA is more important than the stem loop (Zetsche et al., 2015; Dong et al., 2016); indeed, conserved DR stem-loops are functionally interchangeable between most members of the cpf1 family (Zetsche et al., 2015), while pre-crRNA might be more important than mature crRNA for targeting plant genes. Xu et al. (2016) observed that LbCpf1 transformed with pre-crRNA can enable higher mutation efficiency than the use of mature crRNA in rice (Xu et al., 2016).

Significantly, research has shown that off-target effects can largely be minimized without sacrificing their on-target counterparts when the CRISPR-Cpf1 system is used rather than CRISPR-Cas9 (Kim et al., 2016; Kleinstiver et al., 2016). Results show that Cpf1 tolerates single or double mismatches in the 3′ PAM-distal region, rather than in the 5′ PAM-proximal region (Fonfara et al., 2016; Kim et al., 2016). In addition, Cpf1 preferentially makes deletions as opposed to insertions (Kim et al., 2016). Although simplified Cpf1 enables more convenient GET, challenges remain in on-target cleavage efficiency as this is lower than the better studied Cas9 (Fonfara et al., 2016; Kim et al., 2016).

## The CRISPR-C2c1 system

The newly-identified RNA-guided endonuclease, C2c1, belongs to the type V-B CRISPR-Cas system (Shmakov et al., 2015; Yang H. et al., 2016; Liu et al., 2017); Figure 1B). The CRISPR-C2c1 system is composed of the ordered c2c1-cas4-cas1-cas2-CRISPR array, similar to the CRISPR-Cpf1 system with the exception of the fact that cas4-cas1 is a fusion protein (Makarova et al., 2011; Shmakov et al., 2015). Indeed, C2c1 is a large protein that comprises between 1,100 and 1,500 amino acids, distinct from any other class 2 effectors (Shmakov et al., 2015). The structure of C2c1 consists of a bi-lobed architecture that resembles a “Crab Claw” consisting of two lobes, an α-helical recognition (REC) lobe that comprises REC1 (helical-I) and REC2 (helical-II), and a NUC lobe that includes wedge (WED, or OBD), RuvC, and Nuc domains (Yang L. et al., 2016; Liu et al., 2017). As a result, C2c1 consists of just a RuvC endonuclease domain and lacks a similar HNH region; RuvC is the most conserved domain of the C2c1 protein, responsible not only for DNA cleavage, but also for sgRNA binding (Liu et al., 2017). In contrast, Nuc is one of the least conserved regions of the C2c1 family and is essential for DNA cleavage activity (Liu et al., 2017). Research by Liu et al. (2017) has shown that the crystal structure of AacC2c1 has 3.1A° resolution when bound to a crRNA-tracrRNA, crRNA is located in the central channel of C2c1, and tracrRNA in positioned in an external surface groove (Liu et al., 2017). This crRNA-tracrRNA duplex can be simplified into chimeric sgRNA, just as is the case in the CRISPR-Cas9 system (Shmakov et al., 2015). Similarly, the 5′-TTN-3′ PAM sequence is analogous to Cpf1, but this system can recognize target DNA in the absence of a PAM-interacting domain (Liu et al., 2017).

This CRISPR-C2c1 system was first shown to be useful for efficient dsDNA cleavage in *Alicyclobacillus acidoterrestris* and subsequently in *Bacillus thermoamylovorans* (Shmakov et al., 2015). C2c1 is a dual-RNA-guided DNA endonuclease rather than being just single-RNA-mediated, as discussed above in the case of Cpf1 (Zetsche et al., 2015; Liu et al., 2017). C2c1 is able to bind sgRNA to form a binary complex and further target DNAs as ternary complexes, which results in staggered breaks (Yang H. et al., 2016; Liu et al., 2017). This cleavage site is known to occur between 14 and 17 bp upstream of the PAM on the target strand and 23 bp upstream of the PAM on the non-target strand; thus, C2c1 results in a staggered DSB with a six-to-eight nt 5′ overhang that facilitates the NHEJ repair mechanism (Liu et al., 2017). It is also noteworthy that because C2c1 is a metal and temperature-dependent endonuclease, highest cleavage activity is seen in the presence of Mn^2+^ rather than Ni^2+^ or Zn^2+^, and that temperature between 37 and 60°C are conducive for the most efficient cleavage activity (Shmakov et al., 2015; Yang L. et al., 2016; Liu et al., 2017). In addition, the base-pairing between crRNA repeats and tracrRNA anti-repeats are important for DNA cleavage because single or double mutations directly reduce cleavage efficiency. More importantly, most single-nucleotide mutants of the 18 nt proximal to the PAM sequence in gRNA are able to cease cleavage activity, while the other 2 nts distal to the PAM sequence are only able to reduce activity (Liu et al., 2017). These data provide clear evidence that C2c1 may be an ideal GET for minimal off-target cleavage activity.

## RNA-dependent RNA cleavage systems

The most in-depth RNA-dependent RNA cleavage technique current available is RNAi. This method was first proposed by Fire et al. (1998) because potent gene interference was observed when dsRNA was injected into cells of *Caenorhabditis elegans* (Fire et al., 1998). This approach was later shown to be a credible, efficient, and specific GET for both mammals and plants (Elbashir et al., 2001; Brummelkamp et al., 2002; Travella et al., 2006). Specifically, the mechanism of RNAi mediated gene silencing comprises three stages, the first of which is initiation, where dsRNA is recognized and cleaved by dicer (a RNAseIII-like enzyme) into short segments of 21–23 nt interfering RNA (siRNA). The presence of 2 nt 3′ overhangs and 5′-phosphate termini are essential for the function of siRNA (Zamore et al., 2000). The second stage, effect, involves the combination of siRNA and assembly with an RNA-induced silencing complex (RISC). These RISCs recognize target mRNA, while an anti-sense siRNA strand is paired next to target mRNA via Watson-Crick base pairing. At the same time the sense siRNA strand is released and the entire combined region is cleaved by the RISC (Dzitoyeva et al., 2001). In the final stage, cascade amplification, dsRNA is amplified with templates of target mRNA, primers of siRNA and RNA-dependent RNA polymerase. To induce more siRNA, the effect stage is repeated over and over again so as to silence the target gene. However, RNAi knockdown means that the DNA sequence diversifies, and knock-out neither occurs completely or permanently.

The latest technique to be developed by Abudayyeh et al. (2016) is C2c2 which enables genome editing at the RNA level (Shmakov et al., 2015; Abudayyeh et al., 2016; Figure 1C). Indeed, similar to CRISPR-Cas9, C2c2 is a single effector type VI CRISPR-Cas protein that was extracted from the bacterium *Leptotrichia shahii*. However, unlike the types discussed above, C2c2 is specific for a SSB in the target gene and contains two conserved R (N) xxxH motifs, as is typical of the higher eukaryote and prokaryote nucleotide-binding domain (HEPN; Grynberg et al., 2003; Anantharaman et al., 2013). In addition, the HEPN superfamily contains several RNase domains, which can enable further construction of an RNA-dependent cleavage system in bacteria (Kwon et al., 2013). The pre-crRNA in C2c2 can be processed to mature crRNA and further loaded into the C2c2 protein to a mixed α/β secondary structure (Grynberg et al., 2003). Taking into account all these factors, Gao et al. (2016) pointed out that C2c2, as an endoribonuclease, is able to cleave any ssRNA given guidance from a specific 28 nt sequence in crRNA alongside HEPN domains which act as dimers (Gao et al., 2016). Thus, the GET of C2c2 enables a new way to regulate the expression of specific genes and to edit specific effectors at the RNA level.

While C2c2 is a single effector endoRNase which can generate ssRNA cleavage following crRNA, HEPN and crRNA domains are the indispensable elements for cleavage events. The secondary structure of LshC2c2-crRNA is able to bind and target the exposed region of ssRNA as well as having a preference for uracil residues (Abudayyeh et al., 2016). To date, high efficiency cleavage of LshC2c2-crRNA has been achieved using spacer lengths of at least 22 nt as well as the presence of stem-loop structure in the DR. Unlike PAM, however, C2c2 has a bias for A, U, or C in the 3′ protospacer flanking site, while DR is essential for maintenance of the stem loop at an optimum 24 nt length. In contrast, both HEPN domains contain two conserved arginine and histidine residues, which facilitate interactions with LshC2c2 and lead to RNA cleavage.

Another recently developed strategy for transcriptional regulation is the use of nuclease-dCas9. This system loaded with either a repressor or an activator that acts on gene transcription leading to a loss of DNA strand cleavage ability (Cheng et al., 2013; Mali et al., 2013c; Lawhorn et al., 2014; Figure 3C). Perez-Pinera et al. (2013), for example, showed that this system is feasible when a VP64 activator is fused to dCas9. This approach led to robust expression of the IL1RN gene in embryonic kidney 293T cells (Perez-Pinera et al., 2013), while Gilbert et al. (2013) demonstrated 93% repression of GFP reporter gene expression when dCas9 was fused with a Krüppel-associated box repressor in human cells (Gilbert et al., 2013). Fusion of EGFP with dCas9 has led to an improved understanding of both the conformation and dynamics of chromosomes at the molecular level (Chen et al., 2013).

## The utilization of gets in plants

GETs have been widely applied across a range of fields, including for the modification of morphology, enhancement of resistance, and the improvement of physiological mechanisms that are associated with plant growth and development. All of these characteristics can be modified via genome editing involving deletions, insertions, and replacements leading to losses, gains, or changes in the function of target genes.

## Loss-of-function via knock-out techniques

Loss-of-function is the simplest and most widespread mechanism currently applied to gain a better understanding of gene function. To achieve this, knock-out techniques are used as powerful tools to alter specific DNA sequences for the analysis targeted genes. In plants, a number of important agronomical traits can be improved via site-directed mutagenesis (Table 1). For example, the ABA-INSENSITIVE4 (ABI4) gene that regulates the expression of ABA was disrupted in *Arabidopsis* using ZFN. This generated abi4 mutants that were insensitive to ABA and exhibited enhanced stress tolerance to higher glucose concentration (Osakabe et al., 2010). In other work, the locus for mildew resistance (TaMLO), responsible for synthesizing a protein enabling resistance to powdery mildew in plants, was knocked-out by TALEN-induced mutagenesis in allohexaploid bread wheat. This resulted in an allozygote TaMLO gene that imparted higher resistance to powdery mildew disease, further analysis has even shown that homozygous mutations can be transmitted to the next generation (Wang et al., 2014). Following knock-out of the bentazon sensitive lethal (OsBEL) gene, which imparts sensitivity to bentazon and sulfonylurea, 41.2% LbCpf1-induced mutations were identified accompanied with bentazon-resistance (Xu et al., 2016). Finally, RBSDV-resistant plants were obtained under infection pressure in the field when a S7-2-RNAi or S8-RNAi vector was transformed into rice, leading to an overall product improvement (Ahmed et al., 2016).

**Table 1:** **Loss-of-function genome deletion results**.

**Species**	**GETs**	**Target gene**	**Mutated phenotype**	**On-targets**		**Detection method**	**Transformation technique**	**Expression system**	**References**
				**HR %**	**NHEJ %**				
*Brassica napus*	RNAi	GPAT4	Abnormal development of several reproductive organs and reduced seed set	N/A		PCR	*Agrobacterium*-mediated transformation	Stable transgenic	Chen et al., 2014
*Solanum lycopersicum*	RNAi	Rep/CP	High level resistance against RBSDV	N/A		PCR + Southern hybridization	*Agrobacterium*-mediated transformation	Stable transgenic	Ammara et al., 2015
*Oryza sativa*	RNAi	S7-2/S8	Allyl alcohol resistance/lack of anthocyanins in the seed coat	N/A		PCR	*Agrobacterium*-mediated transformation	Stable transgenic	Ahmed et al., 2016
*Arabidopsis thaliana*	ZFN	ADH1, TT4	Allyl alcohol resistance/lack of anthocyanins in the seed coat		7/16	PCR + sequencing	Floral-dip transformation	Stable transgenic	Zhang et al., 2010
*Arabidopsis thaliana*	ZFN	ABI4	ABA and glucose insensitivity		3	Surveyor nuclease assay	*Agrobacterium*-mediated transformation	Stable transgenic	Osakabe et al., 2010
*Glycine max*	ZFN	DICER-LIKE (DCL)	Large bulbous internodes		3	PCR method	*A.rhizogenes* hairy-root transformation	Stable transgenic	Curtin et al., 2011
*Solanum lycopersicum*	TALEN	PRO	Taller and had lighter green leaves with smoother margins		15	PCR-RE	*Agrobacterium*-mediated transformation	Stable transgenic	Lor et al., 2014
*Triticum aestivum*	TALEN	TaMLO	Resistance to powdery mildew		23–38	PCR-RE	Particle bombardment	Stable transgenic	Wang et al., 2014
*Oryza sativa*	TALEN	OsBADH2	Non-fragrant rice		30	PCR-RE	Particle bombardment	Stable transgenic	Shan et al., 2015
*Oryza sativa*	TALEN	Os11N3	Resistance to bacterial blight		48	PCR + sequencing	*Agrobacterium*-mediated transformation	Stable transgenic	Li et al., 2012
*Zea mays*	TALE	*Zm*IPK	Reduces phytic acid content in maize seeds		9	PCR/RE + sequencing	*Agrobacterium*-mediated transformation	Transient expression	Liang et al., 2014
	CRISPR/Cas9				13				
*Zea mays*	CRISPR/Cas9	LIG1	Leaf wih ligule		91	QPCR + sequencing	Biolistic-mediated transformation	Transient expression	Svitashev et al., 2015
		Ms26	Male sterile		77				
		Ms45			100				
		ALS1	Resistance to chlorsulfuron		1.3/0.03				
		ALS2			2.5/2.7				
*Glycine max*	CRISPR/Cas9	DD20	N.A	4.6	59.3	QPCR assay	Particle bombardment	Stable transgenic	Li et al., 2015
		DD43	N.A	3.8	76				
*Arabidopsis thaliana*	CRISPR/Cas9	TRY, CPC, ETC2	Upwardly curled leaves		10	T7E1	Floral-dip transformation	Stable transgenic	Xing et al., 2014
*Solanum lycopersicum*	CRISPR/Cas9	SlAGO7	Compound flat leaves become needle like or wiry		48	PCR + sequencing	*A. tumefaciens*-mediated transformations	Stable transgenic	Brooks et al., 2014
*Oryza sativa*	CRISPR/Cas9	OsPDS (SP1)	Albino and dwarf		15	PCR /RE+sequencing	PEG-protoplast transfection	Transient expression	Shan et al., 2013
		OsPDS (SP2)		6.9					
*Oryza sativa*	CRISPR/Cas9	CAO1	Pale green leaf	13.3	70	PCR + sequencing	Agro-transformation of callus	Stable transgenic	Miao et al., 2013
		LAZY1	Tiller-spreading	50	41.7				
*Oryza sativa*	CRISPR/Cas9	ROC5	Rice Outermost Cell-specific gene5		26	PCR + RE	Agro-transformation of callus	Stable transgenic	Feng et al., 2013
		SPP	Stromal Processing Peptidase		5				
		YSA	Albino leaf phenotype		48–75				
*Arabidopsis thaliana*		BRI1	Retarded growth and rolling leaves		26–33	PCR + RE	Agro-transformation by floral dip	Stable transgenic	
		JAZ1	Jasmona-zim-domain protein1		47				
		GAI	Dwarf phenotype		25				
*Arabidopsis*	CRISPR/Cas9	*mir169a*	Improved drought resistance		20	PCR + sequencing	*Agrobacterium*-mediated transformation	Stable transformation	Zhao et al., 2016
*Triticum aestivum*	CRISPR/Cas9	inox	N.A		17.9–22.3	PCR + sequencing	Agro-transfection of immature embryos	Transient expression	Upadhyay et al., 2013
		pds	N.A		18.4–22.3				
*Nicotiana benthamiana*	CRISPR/Cas9	pds			1.8	PCR + sequencing	Agro-infiltration of leaves	Transient expression	
*Triticum aestivum*	CRISPR/Cas9	TaMLO	N.A		28.5	PCR + RE	PEG-protoplast transfection	Transient expression	Shan et al., 2013
*Arabidopsis thaliana*	CRISPR/Cas9	PDS	Leaves with photobleached phenotype	2.70		PCR /RE+sequencing	*Agrobacterium* leaf infiltration	Transient expression	Li et al., 2013
*Nicotiana tabacum*				4.8					
*Oryza sativa*	CRISPR/Cpf1	BEL PDS	Resistence to bentazon and sulfonylurea Albino and dwarf		41.2 21.4	T7E1 assay	*Agrobacterium*-mediated transformation	Stable expression	Xu et al., 2016

Some phenotype-related characteristics can also be improved by using fixed-point knock-out techniques. For example, phytoene desaturase gene (PDS) loss-of-function mutations were obtained in T2 bread wheat using RNAi (Travella et al., 2006), Ospds disruption strains induced by CRISSPR/Cas9-meditated mutagenesis have been identified in rice, and albino and dwarf phenotypes are seen as expected (Shan et al., 2013). Similar phenotypes have also been observed in the case of CRISPR-Cas9-induced mutagenesis in *A. thaliana* and *N. tabacum* (Li et al., 2013), while 21.4% Ospds mutations were generated using LbCpf1 system (Xu et al., 2016). A 13.3% proportion of loss-of-function mutants were generated when the chlorophyll a oxygenase 1 gene (cao1) was edited by CRISSPR-Cas9, these plants exhibited a pale green leaf phenotype due to defective synthesis of chlorophyll b (Chl b; Miao et al., 2013). Similarly, a 50% proportion of loss-of-function mutants to the LAZY1 (LA1) gene showed a tiller-spreading phenotype after tillering stage (Miao et al., 2013). In addition, anthocyanin is the flavonoid-derived metabolite which is beneficial to humans because of its strong antioxidant activities (Hou et al., 2004). Zhang et al. (2010) showed that 7% of transparent testa 4 (tt4) gene mutations achieved by application of ZFN showed a deficiency of anthocyanin in the seed coat (Zhang et al., 2010), while the content of phytic acid in maize seeds was successfully reduced by knocking out ZmIPK (an enzyme involved in phytate biosynthesis) using TALEN and CRISPR-Cas9 (Liang et al., 2014). Finally, Soyk et al. (2016) obtained mutations of the self-pruning 5G gene (SP5G), a paralog of florigen that represses flowering activity by applying CRISPR-Cas9 GET. This approach enabled them to successfully broach the limit of day-length sensitivity and create early-yielding varieties (Soyk et al., 2016). In general, these approaches have demonstrated great potential for maize, wheat, and soybean. As for multiple-gene knock-out, it has also been conducted, for example, try cpc etc2 triple mutants that display upwardly-curled leaves as would be expected in *Arabidopsis* (Xing et al., 2014).

## Gain-of-function via knock-in techniques

In addition to knock-out in plants, it is also possible to integrate genes with potential applications into the genome to modify specific phenotypic traits (Table 2). For example, phosphorus pollution and herbicide resistance are huge current problems; herbicide tolerant and phytate accumulation-mitigated *Zea mays* mutants have been obtained by knocking-out the IPK1 gene and simultaneously introducing a new herbicide-tolerance gene via ZFN (Shukla et al., 2009). Research by Cai et al. (2009) has shown that a phosphinothricin phosphotransferase herbicide resistance gene can be integrated into the endochitinase gene of tobacco via HR using ZFN (Cai et al., 2009). Schiml et al. (2014) demonstrated that the NPTII gene which confers kanamycin resistance can be accurately inserted into the AtADH1 of *A. thaliana* (Schiml et al., 2014), while Li et al. (2015) revealed that hygromycin resistance gene can be conferred into soybean via HR-mediated integration of CRISPR-Cas9 (Li et al., 2015). Besides, Svitashev et al. (2015) showed that bialaphos resistance gene can be integrated into maize via CRISPR-Cas9 (Svitashev et al., 2015). Reporter genes inserted into pre-selected sites will also enable a better understanding of the expression levels of targeted genes accompanied by dynamic changes. For example, Zhang et al. (2013) delivered TALEN plasmids that incorporated yellow fluorescent protein (YFP) and single-strand annealing (SSA) reporters into tobacco protoplasts. This work showed that 14% of targeted YFP insertions were successful (Zhang et al., 2013), while Wang et al. (2014) integrated GFP into TaMLO using TALEN to generate 6.5% green florescence in wheat protoplasts. Further study revealed that these insertions can stabilize inheritance to T1 populations in a Mendelian fashion (Wang et al., 2014). These findings make the introduction of exogenous DNA fragments into plant genes possible.

**Table 2:** **Gain-of-function genome insertion results**.

**Species**	**GETs**	**Target gene**	**Gain-of-function**	**On-targets**		**Detection method**	**Transformation technique**	**Expression system**	**References**
				**HR**	**NHEJ**				
*Zea mays*	ZFN	IPK1	Herbicide-tolerance	18.6%		Deep sequencing	Whisker-mediated transformation	Stable transgenic	Shukla et al., 2009
*Nicotiana tabacum*	ZFN	MEL1	Pat herbicide resistance gene cassette	10%		Nested PCR	*Agrobacterium* transformation	Stable transgenic	Cai et al., 2009
*Nicotiana tabacum*	TALEN	ALS (SurA, SurB)	YFP		14%	SSA assay	PEG-protoplast transfection	Transient expression	Zhang et al., 2013
*Triticum aestivum*	TALEN	TaMLO	Protoplasts with GFP		6.5%	PCR-RE	Particle bombardment	Stable transgenic	Wang et al., 2014
	CRISPR/Cas9	TaMLO	His-tag insert to TaMLO-A1/ Myc-tag insert to TaMLO-B1		1/69//3/39	PCR+sequencing	Particle bombardment	Stable transgenic	
*Oryza sativa*	CRISPR/Cas9	PDS	Single-stranded oligo with a KpnI + EcoRI site	6.8%		PCR /RE+sequencing	PEG-protoplast transfection	Transient expression	Shan et al., 2013
*Arabidopsis thaliana*	CRISPR/Cas9	ADH1	Kanamycin resistance genes-NPTII	14.6%		PCR +Southern blotting	*Agrobacterium*-mediated transformation	Stable transgenic	Abudayyeh et al., 2016
*Glycine max*	CRISPR/Cas9	DD20	Hygromycin resistance	15.5%	57.1%	QPCR assay	Particle bombardment	Stable transgenic	Li et al., 2015
		DD44		2.2%	2.2%				
*Zea mays*	CRISPR/Cas9	LIG	Resistant to bialaphos	4.1%^*^	86%^*^	PCR+sequencing	Particle bombardment	Transient expression	Svitashev et al., 2015
				0	84%		*Agrobacterium*-mediated transformation	Stable transgenic	

* *The frequency obtained when gRNA, Cas9, and donor DNA all in a single vector*.

## Change-of-function via gene replacement

Gene replacement can be achieved when an extraneous donor is available that possesses a similar terminal with respect to targeted genes. Thus, gene replacement can only be stimulated by DSB when an external DNA donor is available with a homologous terminal to the targeted DNA. When this is the case, this approach can facilitate accurate DNA repair mechanisms and modify genes for a new phenotype or function (Table 3).

**Table 3:** **Change-of-function genome replacement results**.

**Species**	**GETs**	**Target gene**	**Changed function**	**On-targets**		**Detection method**	**Transformation technique**	**Expression system**	**References**
				**HR %**	**NHEJ %**				
*Arabidopsis*	ZFN	QQR-ZFN	GFP to hygromycin-resistant		4.80	PCR + sequencing	*A. tumefaciens*-mediated transformation	Stable transgenic	Weinthal et al., 2013
*Nicotiana tabacum*					6.70				
*Arabidopsis*	ZFN	PPO-loss	Sensitive to the herbicide butafenacil	2		PCR+ Southern blot analysis	*Agrobacterium*-mediated floral dip transformation	Stable transgenic	de Pater et al., 2013
*Nicotiana tabacum*	ZFN	SurA, SurB	Resistance to imidazolinone and sulphonylurea herbicides	N.A		Pyrosequencing	Electroporation	Stable transgenic	Townsend et al., 2009
*Nicotiana tabacum*	TALEN	ALS (SurA, SurB)	Herbicide resistance	4		Protoplast-based single-strand annealing assay	PEG-protoplast transfection	Transient expression	Zhang et al., 2013
*Oryza sativa*	TALEN	OsALS	Non-herbicides resistance to bispyribac-sodium resistant	6		PCR+sequencing	Ballistic bombardment	Stable transgenic	Li et al., 2016
*Pyricularia oryzae*	CRISPR/Cas9	SRS2	Bialaphos resistance gene	100.00		PCR analysis	PEG-protoplast transfection	Transient expression	Arazoe et al., 2015
		SDH genescytalone dehydratase	Bialaphos-resistant with white phenotype	36.1–83.6					
*Nicotiana tabacum*	CRISPR/Cas9	PDS	NbPDS locus to AvrII site	10.7		PCR + sequencing	*Agrobacterium* leaf infiltration	Transient expression	Li et al., 2013
*Arabidopsis*	CRISPR/Cas9	*At*TFL1	AtTFL1 to eGFP	0.80		PCR + sequencing	*Agrobacterium*-mediated transformation	Stable transgenic	Zhao et al., 2016
*Glycine max*	CRISPR/Cas9	Avr4/6	Avr4/6 gene to NPT II gene	13.20		PCR + sequencing	PEG-mediated protoplast transformations	Transient expression	Fang and Tyler, 2016

Weinthal et al. (2013) co-delivered an acceptor DNA molecule (GFP) and a donor DNA molecule (a promoter-less hygromycin B phosphotransferase-encoding gene) flanked by ZFN recognition sequences into *N. tabacum* and *A. thaliana*. The results of this study showed that GFP coding sequences were completely removed along with the recovery of hyp-resistance (Weinthal et al., 2013). In addition, the acetolactate synthase gene (ALS), a major enzyme for herbicides development which can tolerate bispyribac-sodium when two crucial positions of W548L and S627I are mutated, was replaced by a DNA donor of OsALS mutations with double point mutations through TALEN-based HR in rice. This study showed that up to 6% bispyribac-sodium-resistant rice lines can be generated and are heritable to the T1 generation with normal morphology (Li et al., 2016). In addition, Fang and Tyler (2016) designed a NPT II-based CRISPR-Cas9 cassette flanked by Avr4/6 that comprised three different lengths of 5′/3′ arm sequences (i.e., 250 bp, 500 bp, and 1 kb); the results of this study showed that the longer the attached flanking sequences are, the higher the frequency of induced HR will be. This further corroborates the success of the replacement of Avr4/6 with the NPT II gene (Fang and Tyler, 2016). Zhao et al. (2016) introduced an eGFP expression cassette flanked by *A. thaliana* terminal flower 1 (AtTFL1) left/right homologous arm into a targeted region of this gene via CRISPR-Cas9-meditated replacement; they found that the frequency of 0.8% stably transformed T0 transgenic plants carried eGFP signal and expressed actively in both the leaves and roots of T1 generations (Zhao et al., 2016). Finally, Li et al. (2013) verified HR-induced gene replacement using CRISPR-Cas9 to show that a frequency of 9.0% AvrII incorporation can be achieved when supplying a dsDNA donor that contained a unique AvrII site flanked by left and right homology arm to the NbPDS locus in *N. benthamiana* protoplasts (Li et al., 2013).

## Conclusions

In sum, GETs have great potential for the future analysis of gene function, including for the targeted therapy of human diseases and crop breeding. Stroud et al. (2016), for example, have argued that that GETs coupled with proteomics present powerful tools for studying mitochondrial disease at the cellular level (Stroud et al., 2016). We therefore expect that these technologies will accelerate progress in molecular directional breeding leading to improvements in the quality of cereals (e.g., rice and wheat) and oil crops (e.g., soybean and oilseed rape). Conventional breeding technologies including the use of chromosome-doubling via the application of colchicine and plasmogamy via PEG are limited to single-species applications. However, given the increasing attention being paid to transgenic food, caution must be exercised in the application of GETs, especially in the case of humans and crops. Taken together, some prerequisites should be followed with regard to both the beneficial and detrimental impacts of GETs on humans. In the first place, potential risks of GETs should be estimated as we move from the laboratory to the field. As unavoidable off-target affects will occur to different degrees, measures such as the analysis and minimization of unfavorable consequences should be taken, recorded, and publicly documented. In addition, the tools used as components of GETs (e.g., ZFN, TALE, and CRISPR) may also have potential cytotoxicity. Secondly, each project that is carried out in this field should encompass a baseline of relative relationships. Third, all satisfactorily-generated transgenic crops or food must be registered and management of this market should be standardized.

## Author contributions

KZ carried out the analysis and wrote the manuscript. NR and BZ made helpful suggestions to the manuscript. ML designed, led and coordinated the overall study.

### Conflict of interest statement

The authors declare that the research was conducted in the absence of any commercial or financial relationships that could be construed as a potential conflict of interest.
